# Enhancement of Inhibition of the *Pseudomonas* sp. Biofilm Formation on Bacterial Cellulose-Based Wound Dressing by the Combined Action of Alginate Lyase and Gentamicin

**DOI:** 10.3390/ijms24054740

**Published:** 2023-03-01

**Authors:** Magdalena Charęza, Katarzyna Przygrodzka, Anna Żywicka, Bartłomiej Grygorcewicz, Peter Sobolewski, Sylwia Mozia, Marcin Śmiglak, Radosław Drozd

**Affiliations:** 1 Department of Microbiology and Biotechnology, Faculty of Biotechnology and Animal Husbandry, West Pomeranian University of Technology in Szczecin, 45 Piastów Avenue, 71-311 Szczecin, Poland; 2 Department of Laboratory Medicine, Pomeranian Medical University in Szczecin, Powstańców Wielkopolskich 72, 70-111 Szczecin, Poland; 3 Department of Polymer and Biomaterials Science, Faculty of Chemical Technology and Engineering, West Pomeranian University of Technology, Piastów 45, 70-311 Szczecin, Poland; 4 Department of Inorganic Chemical Technology and Environment Engineering, Faculty of Chemical Technology and Engineering, West Pomeranian University of Technology in Szczecin, ul. Pułaskiego 10, 70-322 Szczecin, Poland; 5 Poznan Science and Technology Park (PPNT), Rubiez 5, 61-612 Poznan, Poland

**Keywords:** bacterial cellulose, alginate lyase, biofilm eradication, antibiotic susceptibility

## Abstract

Bacterial biofilms generally contribute to chronic infections, including wound infections. Due to the antibiotic resistance mechanisms protecting bacteria living in the biofilm, they are a serious problem in the wound healing process. To accelerate the wound healing process and avoid bacterial infection, it is necessary to select the appropriate dressing material. In this study, the promising therapeutic properties of alginate lyase (AlgL) immobilised on BC membranes for protecting wounds from *Pseudomonas aeruginosa* infection were investigated. The AlgL was immobilised on never dried BC pellicles via physical adsorption. The maximum adsorption capacity of AlgL was 6.0 mg/g of dry BC, and the equilibrium was reached after 2 h. The adsorption kinetics was studied, and it has been proven that the adsorption was consistent with Langmuir isotherm. In addition, the impact of enzyme immobilisation on bacterial biofilm stability and the effect of simultaneous immobilisation of AlgL and gentamicin on the viability of bacterial cells was investigated. The obtained results showed that the AlgL immobilisation significantly reduced the amount of polysaccharides component of the *P. aeruginosa* biofilm. Moreover, the biofilm disruption by AlgL immobilised on BC membranes exhibited synergism with the gentamicin, resulting in 86.5% more dead *P. aeruginosa* PAO-1 cells.

## 1. Introduction

The world is struggling with the growing phenomenon of antibiotic resistance, primarily caused by unchecked and often excessive use of antimicrobials. Despite ongoing research, few new antibiotics have been discovered recently, whereas the phenomenon of antimicrobial resistance continues to grow. Moreover, antibiotics are only effective against microorganisms in the form of planktonic cells, while most bacteria produce protective biofilms that prevent the penetration and effective action of antibiotic molecules into their structure [1,2,3]. *Pseudomonas aeruginosa* is an opportunistic pathogen that causes nosocomial infections that are particularly dangerous for immunocompromised patients. Treatment of such infections with standard antibiotic therapy is often unsatisfactory due to the ability of these bacteria to form structurally complex biofilms composed primarily of exopolysaccharides, eDNA, proteins, and lipids [4,5]. This kind of bacterial biofilms are typically responsible for chronic infections, including wound infections, e.g., pressure, venous leg, and diabetic foot ulcers. They delay the wound healing process, reduce the action of bactericides, and impair the host immune system. A widely used method for removing biofilms from chronic wounds and improving the healing process is the mechanical removal of necrotic tissue and microorganisms. An alternative solution is to administer compounds called adjuvants along with antibiotics. The use of phages (viruses that attack bacterial cells) can also be effective, such as those that can produce enzymes such as polysaccharide depolymerase, which can decompose the extracellular polymeric substances (EPS) matrix, allowing the phages to diffuse into the biofilm and attach to cellular structures, enabling replication [3,6,7]. Additionally, to destabilise the EPS matrix and enhance antibiotic therapy, it is also possible to use other types of enzymes, such as glycosidases, proteases and DNase [8]. A particularly interesting group of enzymes are those that play key roles in the biochemical pathways of biofilm matrix synthesis. These include enzymes such as glycoside hydrolases PelA_h_, PslG_h_, and alginate lyase (AlgL), which, under favourable circumstances, can degrade Pel, Psl, and alginate polymers present in the EPS matrix produced by *P. aeruginosa* strains [9,10,11,12]. In fact, alginate is the most abundant extracellular matrix polysaccharide in the EPS of many bacteria. This polymer consists of β-D-mannuronate (M) and α-L-guluronate (G) as monomeric units that are linked in three different kinds of blocks, poly β-D-mannuronate (polyM), poly α-L-guluronate (polyG) or the heteropolymer (polyM/G) [13,14,15]. Additionally, the alginate produced by *P. aeruginosa* has O-acetyl groups on the C2- and/or C3- position of the β-D-mannuronate residues, which significantly affects the physicochemical properties (e.g., viscosity, ability to bind divalent cations, and water-binding capacity) of the biofilm matrix and the structure of the biofilm. The typically highly hydrated alginate-containing biofilm matrix protects bacterial cells against dehydration and the host immune system. Additionally, the biofilm matrix also contributes to antibiotic resistance mechanisms. Further, it has been observed that antibiotic concentrations below the minimal inhibitory concentration (MIC) value can result in an increase in alginate synthesis by *P. aeruginosa* [16,17]. Additionally, Hatch and Schiller reported that negatively charged alginate could block the bactericidal activity of positively charged antibiotics, such as aminoglycosides and polymyxin B [18]. The biosynthesis of alginate by *Pseudomonas* sp. is coded primarily in a single operon that consists of twelve genes. Among them, the *algL* gene is responsible for encoding alginate lyase (AlgL) [19]. AlgL is an element of the polymer transport apparatus, which moves alginate across the periplasm to porin-like protein AlgE and results in secretion. AlgL also plays an important role in regulating alginate synthesis by degrading excess polysaccharides, which can be toxic to the bacterial cell [14,20,21]. AlgL can degrade alginate via a β-elimination mechanism of the glycosidic bonds to form unsaturated oligosaccharides. Due to their substrate specificity, AlgLs can be distinguished as G block specific, M block specific, or poly M/G specific [22,23]. Importantly, it has been shown that AlgL can effectively inhibit biofilm formation by mucoid *P. aeruginosa* [24] and increases the sensitivity of bacterial cells to various antibiotics [13,25,26]. Thanks to these important properties, many medicinal and pharmaceutical applications based on this enzyme are being developed, including advanced delivery systems based on silver nanocomposite particles or other biopolymers, such as chitosan and hyaluronan, as carriers for AlgL. These solutions have been successfully applied to treat *P. aeruginosa* lung infections [27,28,29,30,31,32]. Of the many biopolymers, bacterial cellulose (BC) is often considered an excellent carrier for various bioactive compounds, including enzymes. Additionally, due to its unique water properties, BC is an ideal material for wound dressing application, tissue regeneration, and as temporary skin substitutes. BC is non-toxic, non-cancerogenic, and biocompatible, plus it has an unusual capacity to retain moisture and can absorb exudates from the injured tissues, accelerating granulation [33]. At the same time, the neutrality of BC in some circumstances can be a disadvantage, and it is typically considered only as a physical barrier for microbes in chronic wound therapy. However, BC could provide much more effective protection against microbial growth if it could be combined with enzymes able to degrade biofilm and/or antibiotics. In fact, using pristine BC as a carrier for enzymes has many advantages, thanks to only a marginal effect on their structure and catalytic properties [34,35,36,37,38]. Despite this, the use of BC as a carrier of AlgL via immobilisation to improve its properties as versatile wound dressing material has not been reported. As a result, the aim of this study was the analysis of the effect of immobilisation of AlgL via physical adsorption on never-dried BC on the stability of biofilm formed by *Pseudomonas* sp. and its susceptibility to aminoglycoside antibiotic gentamicin.

## 2. Results and Discussion

### 2.1. Characteristic of Recombinant AlgL and the Effect of Temperature on the Enzymatic Activity of AlgL

**Figure 1:**
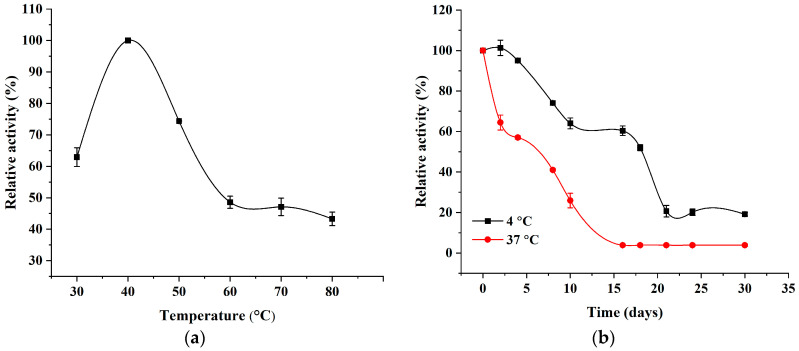
Effects of temperature on the activity (**a**) and time depended on stability at 4 °C and 37 °C (**b**) of soluble AlgL.

The recombinant AlgL with a His_6_ tag at the N- terminus was purified by metal affinity chromatography with Ni^2+^ resin column, and usually, approximate 10 mg/mL recombinant enzyme from litre of bacterial cell culture was obtained with the specific activity of 150.0 U/mg. This result is similar to those reported by Xiao et al., who purified recombinant AlgL from *Pseudomonas* sp. QD03 with a yield of 10.3 mg/L of cultivation medium and a specific activity of 188.5 U/mg [15]. The molecular mass of recombinant alginate lyase determined using SDS-PAGE was 40 kDa and agreed with the value predicted from the amino acid sequence. To determine how long the recombinant AlgL, potentially used in medical dressings, will be stable, both under long-term storage at 4 °C and when applied to the wound at human body temperature, its temperature optimum and stability at 4 °C and 37 °C for 30 days were tested. The effect of temperature on AlgL was analysed within the broad range of the temperature from 30 °C to 80 °C. The optimal temperature for alginate lyase activity was established at 40 °C (Figure 1a). These results were comparable to Ghadam et al., who demonstrated that AlgL has the best enzymatic activity at 37 °C, whereas the enzyme stability at 4 °C and 37 °C at pH 7.4 were studied. After 4 days of storage at 4 °C, AlgL remains at almost 100% of the initial activity [10]. Subsequently, the activity of the enzyme decreases and reaches 20% of initial activity after 21 days and which remains at this level for 30 days. The storage of AlgL at 37 °C caused a decrease in activity by about 70% after 4 days and by about 95% after 21 days, while after 30 days, the enzyme completely lost its activity.

### 2.2. P. aeruginosa PAO-1 Biofilm Inhibition and Disruption of Biofilm Biomass with Alginate Lyase

**Figure 2:**
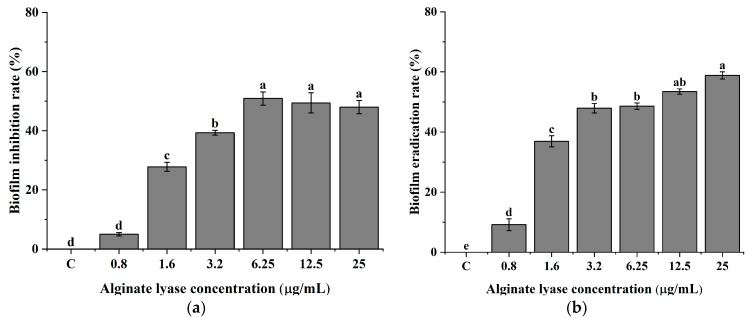
Dose–response of *P. aeruginosa* PAO-1 biofilm formation inhibition (**a**) and disruption of biofilm biomass (**b**). The means with the same superscript are not significantly different, with *p* > 0.05. Error bars represent standard deviation.

To evaluate the potential clinical application of AlgL, the effective inhibition of biofilm formation and eradication of 24 h mature biofilm of *P. aeruginosa* PAO-1 were tested. The effect of AlgL on biofilms was evaluated by crystal violet staining. The prophylactic treatment of *P. aeruginosa* PAO-1 biofilm with AlgL was tested in concentrations from 0.8 μg/mL to 25.0 μg/mL. The enzyme was added at the time of inoculation, and the biofilm was incubated for 24 h at 37 °C. As shown in Figure 2a, the inhibitory effect depended on enzyme dose, and even a small amount of enzyme significantly affected the biofilm formation. The AlgL at a concentration of 0.8 μg/mL did not influence biofilm formation inhibition, while in the range of 1.6 μg/mL and 3.2 μg/mL in 28% and 39%, inhibition of biofilm formation was observed. At the higher concentration of AlgL 6.25 μg/mL, the maximal inhibition (50%) of biofilm formation was observed. However, the further increase of its amount to 25 μg/mL did not have any significant effect. The results for eradicating 24 h mature *P. aeruginosa* PAO-1 biofilm are presented in Figure 2b. The lowest concentration of AlgL resulted in only around 10% of eradication rate, while the concentration of 1.6 μg/mL showed 37%. Application of AlgL concentration from 3.2 to 12.5 μg/mL resulted in 48%, 49%, and 53% biofilm eradication, respectively. However, the best effect was obtained at a concentration of 25.0 μg/mL, where around 60% of biofilm eradication was obtained. According to Tavafi et al., AlgL can inhibit the biofilm formation in the mucoid *P. aeruginosa* infections by more than 97% in the concentration of 9.37 μg/mL and 18.75 μg/mL, while the eradication rate was also more than 97% in the concentration of 18.75 μg/mL and 37.5 μg/mL [39].

### 2.3. Cytotoxicity Study of AlgL

**Figure 3:**
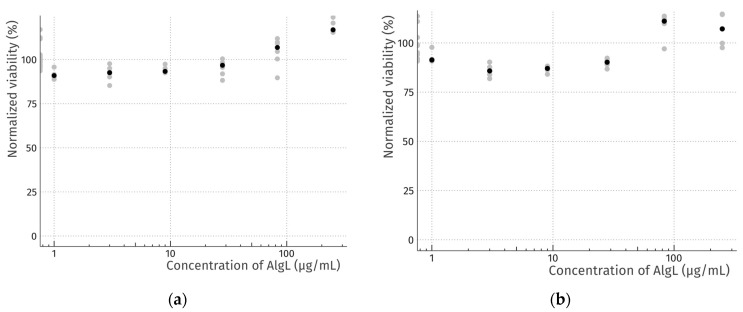
Cell viability of L929 murine fibroblasts exposed to a range of concentrations of recombinant AlgL for 24 h (**a**) and 48 h (**b**). Data were normalised to vehicle control (PBS). Grey dots represent technical replicates, and black dots indicate medians.

**Figure 4:**
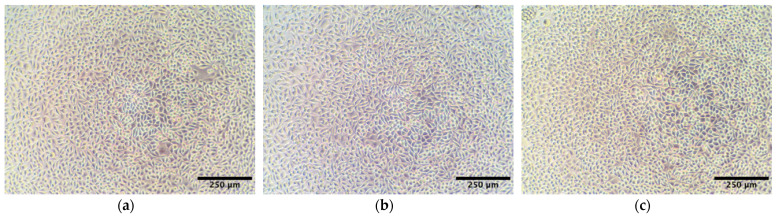
Morphology of L929 cells of non-treated cells (**a**) and incubated with AlgL (250 μg/mL) for 24 h (**b**) and 48 h (**c**).

Ideal wound dressings should be characterised by many features, of which biocompatibility plays one of the most important roles [40]. BC, as a natural polymer, is widely used as a wound dressing material precisely because of its compatibility with living tissue. Introduction to its structure or immobilisation of various types of compounds may, however, cause a cytotoxic effect [41,42]. It has been proven that some enzymes from the group of glycohydrolases with potential use in biofilm eradication may be toxic to mammalian cells [43]. For this reason, a dose–response study of the potential cytotoxicity of recombinant AlgL on mammalian subcutaneous fibroblasts (murine L929 cell line) was examined. The cell viability after exposure to a range of concentrations (1–250 μg/mL) of AlgL for 24 and 48 h was assessed (Figure 3). In both cases, no adverse effect on cell viability was observed using the resazurin assay, and light microscopy confirmed robust growth and normal morphology for all doses (Figure 4). Our results are in line with results from previous studies by Bayer et al., who found that AlgL from *Bacillus circulans* shows no cytotoxic effect when added intravenously to rabbits [44]. On the other hand, this research differs significantly from the findings conducted by Redman et al., who find out the cytotoxicity effect of algae-origin AlgL against fibroblast cell line CCD110 and human epithelial cell line after 3 h treatment [43]. Despite the fact that, in this work, it was found that recombinant AlgL from *Pseudomonas aeruginosa* PAO-1 has no cytotoxic potential, additional research on human cells is necessary to make it suitable for biomedical application.

### 2.4. Immobilisation of AlgL on BC Pellicles

**Figure 5:**
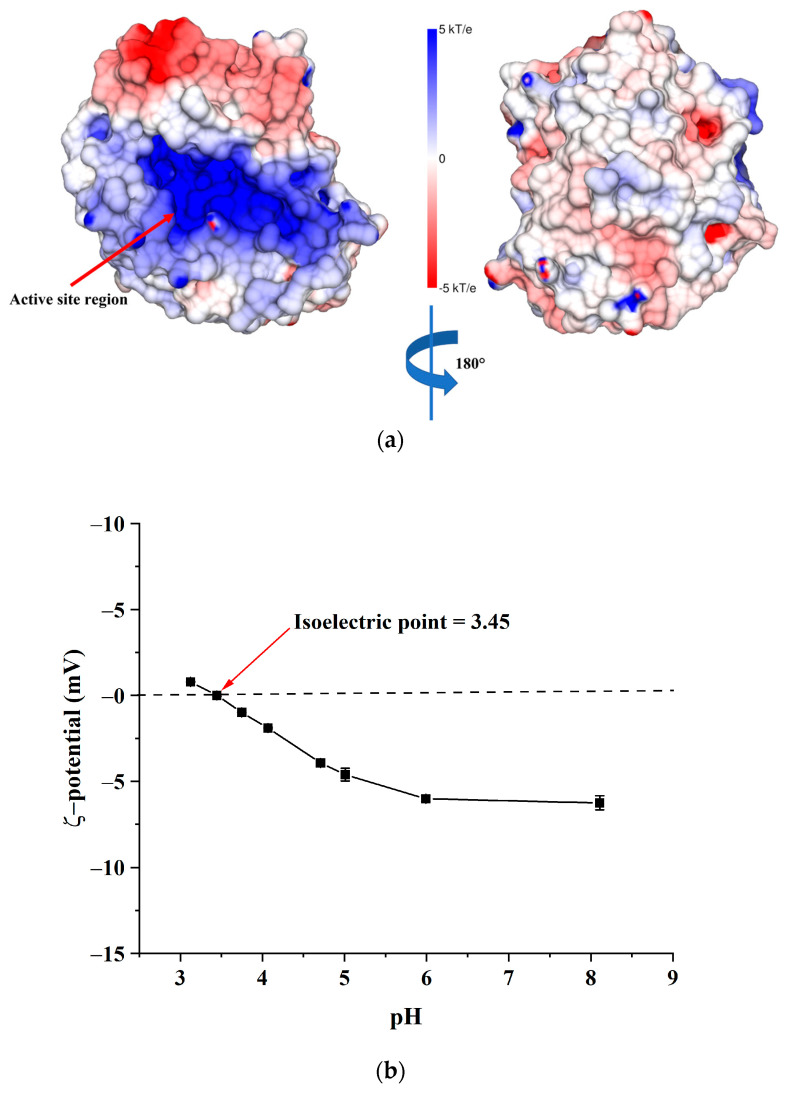
Distribution of electrostatic potential on the molecular surface of AlgL protein (PDB:4OZV) [48] (**a**). The potential was [49] calculated according to the predicted by using the PROPKA server the pK values of titratable amino acid. The electrostatic potential was calculated with the assumption of environment pH 7.4 using an APBS solver via a PDB2PQR server. The results of the calculation were analysed with the UCSF Chimera 1.13.1rc package [50]. The ζ-potential of BC membranes depends on the pH of the solution (**b**).

One of the simplest methods of enzyme immobilisation is adsorption on the surface of the carrier. The adsorption mechanism is based on weak bonds such as Van der Waal’s forces and electrostatic and hydrophobic interactions. The immobilisation by adsorption is an economical process that does not require specialised reagents. Moreover, due to the lack of functionalisation of the carrier and mild conditions of the process, it usually does not significantly influence the enzyme’s activity and stability [45]. Figure 5a shows the electrostatic surface potential of AlgL at pH 7.4. The catalytic active centre of AlgL is characterised by an electropositive potential that is a consequence of accommodation to negatively charged alginate surface coming from the carboxyl group of glucuronic and mannuronic acid [46]. The remaining areas of the protein surface are largely interspersed with both electropositive and electro-negative patches. The ζ-potential of BC depends on the solution pH, and is shown in Figure 5b. BC has positive ζ-potential in an acidic condition at pH 3.0 and negative ζ-potential at pH values from 4.0 to 8.0, with the isoelectric point at pH 3.4 where ζ-potential is equal to zero. The reason for the low isoelectric point and plateau region in the alkaline pH range is the presence of acidic -OH groups on the BC surface. The plateau observed at high pH values is the result of a complete dissociation of the acid functional groups, and thus, the BC surface is negatively charged [47].

**Figure 6:**
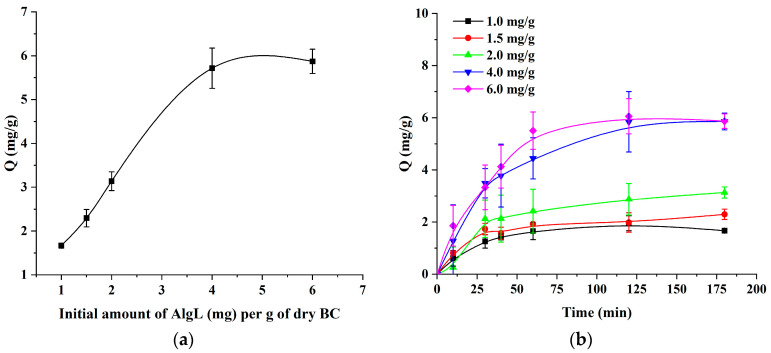
(**a**) The effect of different AlgL concentrations on the adsorption on BC. (**b**) The effect of time on the amount of AlgL adsorbed onto BC.

Figure 6a shows the effect of the initial concentration of AlgL (mg/g of dry BC) on the adsorption on BC membranes at pH 7.4. The results show that the amount of AlgL absorbed increases with an increasing concentration of the protein and then tends to level off. The maximum amount of AlgL absorbed on BC was 6.0 mg/g of dry BC. Figure 6b shows the effect of time on the amount of AlgL adsorbed per gram of BC at pH 7.4. As shown, the adsorption of the AlgL sharply increases during the first 60 min. The equilibrium is reached after 2 h of the adsorption process.

### 2.5. Adsorption Kinetics

**Figure 7:**
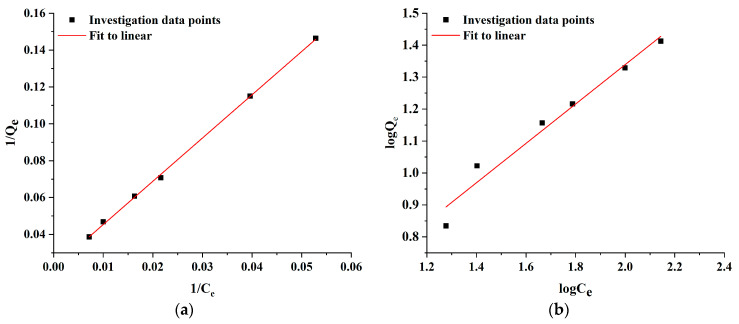
The linear fitting of Langmuir isotherm model (**a**) and Freundlich isotherm model (**b**) for adsorption kinetics.

**Figure 8:**
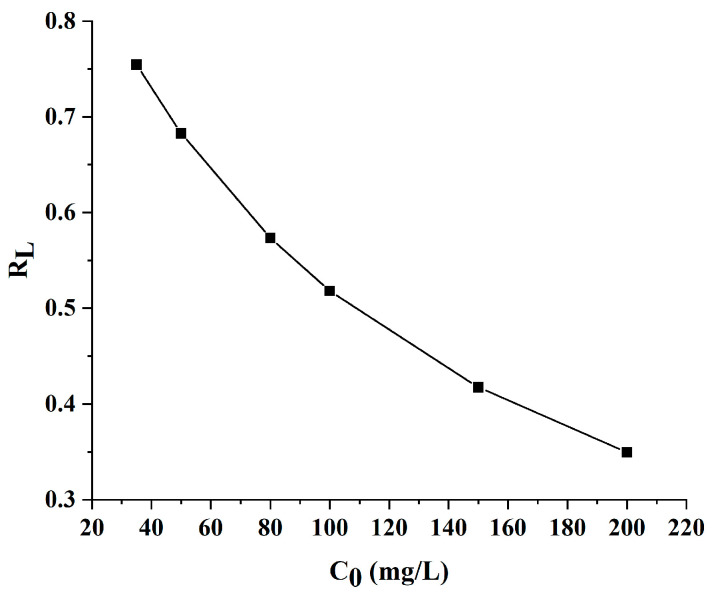
Separation factor R_L_ for the adsorption of AlgL on BC.

To elucidate the mechanism of AlgL adsorption onto BC membranes, the Langmuir and Freundlich isotherm models were utilised. The Langmuir isotherm theory assumed that the adsorbate could form a monolayer of molecules on a homogenous surface and interact with adsorption sites, and the likelihood of increased adsorption increases with the available surface. In contrast to the Langmuir isotherm model, the Freundlich model describes multilayer adsorption on heterogeneous surfaces, and the stronger binding sites on the support are occupied first, and the binding strength decreases with increasing occupied sites [51,52,53]. The adsorption isotherms of AlgL were determined by the adsorption experiment at 25 °C using 50 mM phosphate buffer at pH 7.4 as media. The Langmuir and Freundlich constants and value of R^2^ for the AlgL adsorption on BC membranes are listed in Table 1 while the linear fitting of both adsorption isotherm models is presented in Figure 7. According to these data calculated by the Langmuir isotherm model, the maximum monolayer coverage capacity (q_m_) and the Langmuir isotherm constant (b) were 45.79 mg/g and 0.0009 L/mg, respectively. The R_L_ value for the different concentrations of AlgL immobilised on BC membranes is shown in Figure 8. For all tested AlgL amounts, the R_L_ value is below 1, which indicates that the adsorption was more favourable for a higher initial concentration of AlgL. The affinity between the adsorbate and adsorbent is determined by the separation factor (R_L_); when this value is between 0 and 1, the Langmuir isotherm model is observed to be favourable [54,55]. For the Freundlich isotherm model, the adsorption capacity (K_f_) and adsorption intensity (1/n) were 1.28 (L/g) and 0.62, respectively. The correlation coefficient shown, that the Langmuir isotherm represents a better fit of experimental data than Freundlich isotherm (Table 1) and indicated that the adsorption was the main reason for AlgL immobilisation on BC [56]. 

### 2.6. Analysis of the AlgL Influence on the Biofilm Development on BC Surface by ATR–FTIR

**Figure 9:**
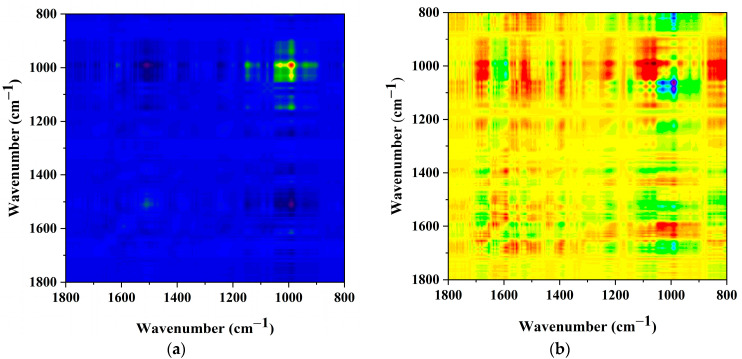
The 2D correlation analysis of the ATR–FTIR spectra with a range of 1800 cm^−1^ to 800 cm^−1^ BC pellicles with immobilised AlgL covered by *P. aeruginosa* PAO-1 biofilm on its surface ((**a**), synchronous; (**b**), asynchronous plots).

The *P. aeruginosa* PAO-1 biofilm formed on BC membranes with immobilised AlgL was analysed using ATR–FTIR. To investigate the detailed differences between the impact of immobilised AlgL on biofilm formation on BC membranes, 2D correlation analysis was applied. The synchronous map (Figure 9a) revealed four major auto-bands on the diagonal position at 990 cm^−1^, 1030 cm^−1^, 1145 cm^−1,^ and 1509 cm^−1^. The most intensive changes in the bands’ intensity were found for the autoband at 990 cm^−1^, followed by 1030 and 1146 cm^−1^ and 1509 cm^−1^. The presence of the band around 990 cm^−1^ is indicative of O–CH_3_ stretching from polysaccharide units. The band at 1030 cm^−1^ is assigned to C–O–C group vibrations in the cyclic carbohydrate structure. The absorption band at 1145 cm^−1^ corresponds to C–O–C stretching vibration from phospholipids and saccharides in the biofilm, while the auto-band with low intensity at 1509 cm^−1^ is assigned to C=O stretching [57,58,59,60,61]. In addition, a set of positive cross bands 990 cm^−1^ vs. 1145 cm^−1^, 1030 cm^−1^ vs. 1145 cm^−1^, 990 cm^−1^ vs. 1030 cm^−1^, 990 cm^−1^ vs. 940 cm^−1^, 990 cm^−1^ vs. 1110 cm^−1^, 990 cm^−1^ vs. 1130 cm^−1^, 940 cm^−1^ vs. 1030 cm^−1^, 990 cm^−1^ vs. 1230 cm^−1^ was visible. The strongest negative cross bands were found for 990 cm^−1^ vs. 1509 cm^−1^, 990 cm^−1^ vs. 1395 cm^−1^, 990 cm^−1^ vs. 1230 cm^−1^, 990 cm^−1^ vs. 940 cm^−1^. The observed bands, especially negative cross bands, indicate on changes in proportion between the polysaccharide components of the biofilm matrix and probably increasing amount of eDNA [62,63]. 

**Figure 10:**
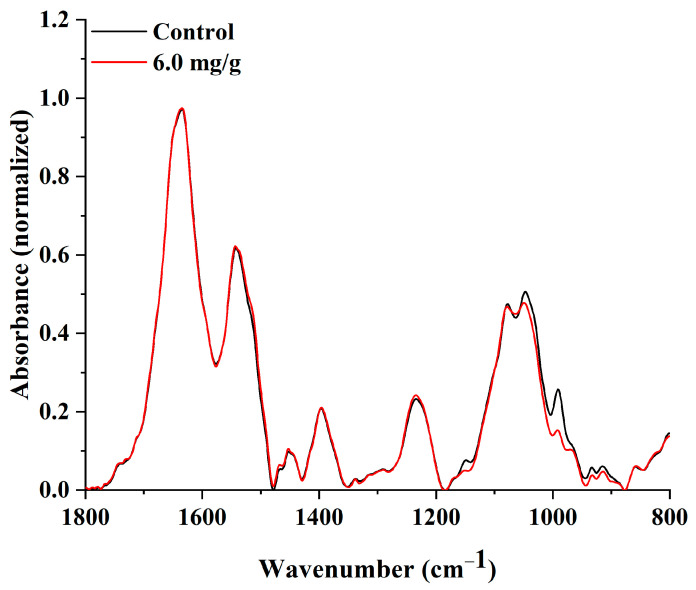
The ATR–FTIR spectra of biofilm of *P. aeruginosa* PAO-1 formed of BC membranes with immobilised AlgL (6.0 mg/g of dry BC) and native BC as a control.

**Figure 11:**
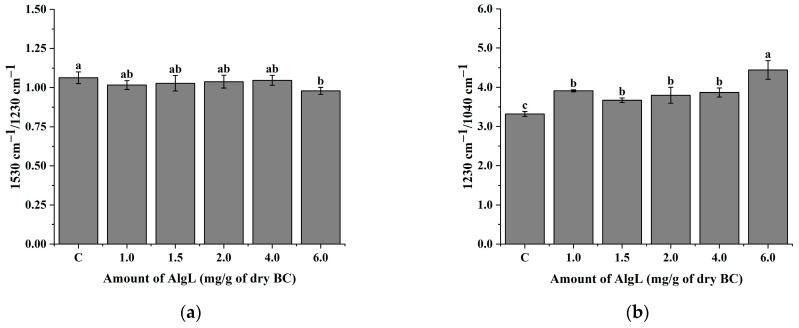
The relative changes in the band ratio, 1530 cm^−1^/1230 cm^−1^ (**a**) and 1230 cm^−1^/1040 cm^−1^ (**b**). The means with the same superscript (a, b) are not significantly different, with *p* > 0.05. Error bars represent standard deviation.

Figure 10 shows the 1D ATR–FTIR spectra of biofilm developed by *P. aeruginosa* PAO-1 on BC membranes with immobilised AlgL (6.0 mg/g of dry BC) and control BC without the enzyme. The spectral region in the wavenumber range of 1800–800 cm^−1^ was examined, which includes spectral bands corresponding to chemical species indicating the significant components of microbial biofilm (protein and nucleic acids ~1600–1180 cm^−1^, polysaccharides ~1200–900 cm^−1^) [12]. The most significant changes were observed in the region between 1200–900 cm^−1^, which can be assigned manly to the symmetric stretching vibration of PO_2^−^_ groups in nucleic acids and a set of bands specific for C-O and C-O-P stretching vibrations of various oligo- and polysaccharides [64,65]. To investigate further possible changes in the protein, lipids, and carboxyl group components of the *P. aeruginosa* PAO-1 biofilm, the ratio between the bands 1530 cm^−1^/1450 cm^−1^ (Figure S1a) and 1230 cm^−1^/1395 cm^−1^ was analysed (Figure S1b). The ratio between the band 1530 cm^−1^, which is assigned to amide II and marking accumulation of biofilm biomass, and the band 1450 cm^−1^ representing CH_2_ bending in lipids show no significant changes between this component of biofilm (Figure S1a). No significant changes were also observed in the case of the ratio between band 1230 cm^−1^ assigned to P=O stretching of >PO^2−^ of phosphodiesters of nucleic acids and 1395 cm^−1^ symmetric stretching C–O of the carboxylate group (COO^−^) (Figure S1b). In contrast, the ratio between the bands 1230 cm^−1^ and 1040 cm^−1^ indicated that the application of AlgL increased the phospholipid, LPS, and nucleic acid content while the polysaccharide content decreased (Figure 11a). Moreover, the band ratio between 1530 cm^−1^ and 1230 cm^−1^, assigned to amide II and amide III, respectively, shows that the immobilisation of AlgL slightly influences on protein and nucleic acids contents in biofilm formed on BC membranes. As shown in Figure 11b, it was observed that the application of the highest tested concentration of AlgL resulted in a slight decrease in the amount of proteins while the amount of nucleic acids increased.

### 2.7. Synergistic Effect of AlgL and Gentamicin

The use of biofilm-dispersing enzymes such as AlgL as an adjuvant which acts by degradation of the extracellular polymer matrix (EPS) surrounding the bacterial cells in biofilm, leads to an increase in their susceptibility to antibiotics and immune cells [66]. As previously reported, the use of AlgL increases the susceptibility of *P. aeruginosa* to various types of antibiotics such as tobramycin, ciprofloxacin [26,67,68], gentamicin, ceftazidime, piperacillin, amikacin [29,69]. From a large variety of different types of antibiotics, aminoglycoside antibiotics, despite the increase in the number of strains showing resistance to at least one clinically used one, are still the most commonly used in the treatment of infections caused by gram-negative bacteria, including *P. aeruginosa.* Unfortunately, high doses of these antibiotics result in the appearance of many toxic effects, such as nephrotoxicity, ototoxicity, or neuromuscular toxicity [70,71,72].

**Figure 12:**
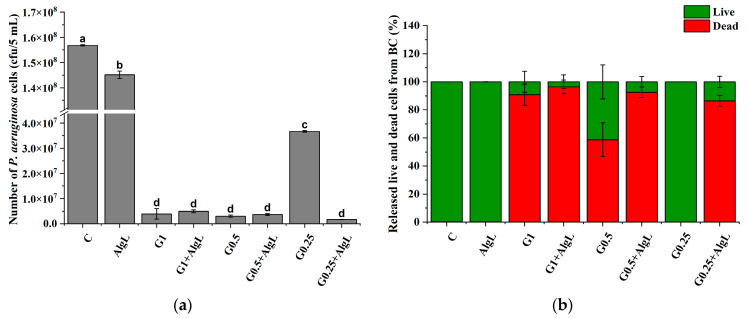
Amount of the *P. aeruginosa* PAO-1 cells (**a**) and the percentage of the live and dead cells of *P. aeruginosa* PAO-1 (**b**) released from BC with immobilised AlgL (0.2 mg/mL) and gentamicin (0.25–1 μg/mL) (**b**). The means with the same superscript (a–d) are not significantly different, with *p* > 0.05. Error bars represent standard deviation.

In these studies, due to the risk of using high doses of aminoglycoside antibiotics the synergistic effects of gentamicin as a representative bactericidal agent immobilised together with AlgL on BC membranes was investigated. The concentration of gentamicin was selected based on the determined MIC value by preparing a wide range of gentamicin concentrations from 1.0 to 0.062 μg/mL. As shown in Figure S2 for *P. aeruginosa* PAO-1, the MIC value of gentamicin was 1.0 μg/mL. For this reason, a concentration equal to 1.0 μg/mL and two concentrations below the MIC value were selected for immobilisation, whereas AlgL concentration was selected according to the results from ATR–FTIR that showed a significant impact of enzyme immobilisation on the EPS structure of biofilm formed on the BC pellicles surface. The total amount of *P. aeruginosa* cells released from BC membranes with immobilised gentamicin and AlgL is shown in Figure 12a. The number of bacterial cells realised from BC with immobilized AlgL was only slightly lower compared to biofilm formed on control BC and any effect on their vitality was not observed Figure 12b. It was noted that the application of gentamicin alone in the concentration of 1.0 μg/mL and 0.5 μg/mL and in combination with AlgL did not show significant changes in the number of *P. aeruginosa* PAO-1 cells, which may suggest that the concentrations of gentamicin used were too high to see any changes. However, in the case of a gentamicin concentration equal to 0.25 μg/mL, a significantly lower number of cells was observed in the case of the combination of enzyme and antibiotic. Therefore, it could be concluded that AlgL disturbs the biofilm matrix development, that results in the reduction of the protective barrier against antibiotics and a visible significant reduction of cell proliferation rate. To further investigate the effect of the enzyme and the antibiotic, the cells were stained with the live–dead method. Figure 12b presents results of live and dead staining of *P. aeruginosa* PAO-1 cells dispersed from biofilm formed on BC. The results show that non-treated samples and samples with immobilised only AlgL did not affect the number of dead cells. Furthermore, the use of gentamicin in the concentration of 1.0 μg/mL in combination with AlgL does not exhibit a statistically significant difference compared to the sample treated with gentamicin only. Whereas after using the gentamicin at a concentration of 0.5 μg/mL and AlgL, it was observed increased over 30% the number of dead *P. aeruginosa* PAO-1 cells compared to the sample treated with gentamicin only. However, the best effect of simultaneous immobilisation of gentamicin and AlgL was observed at a concentration of 0.25 μg/mL of the antibiotic, where the use of the antibiotic alone did not affect the viability of *P. aeruginosa* PAO-1 cells, while in combination with the AlgL, 86.5% dead cells were observed. 

## 3. Materials and Methods

### 3.1. Cloning and Expression of AlgL from P. aeruginosa PAO-1

Recombinant alginate lyase was prepared according to a previously described protocol in [12] used for recombinant PelA_h_ hydrolase with slight modifications. The alginate lyase *algL* cDNA (GenBank ID AAG06935.1) was amplified from the genomic DNA of *P. aeruginosa* PAO-1, with omission predicted by SignalP 5.0 server signal peptide sequence region. The pET28a (Merck KGaA, Darmstadt, Germany) was used as a vector DNA template. Insert and vector was amplified using the primers listed in Table S1 and Phusion High-Fidelity DNA Polymerase (New England Biolabs, Ipswich, MA, USA) by polymerase chain reaction. PCR amplified insert and vector DNA was then treated with DpnI for one hour at 37 °C. Next, the *algL* gene was assembled with the pET28a (Merck KGaA, Darmstadt, Germany) expression vector using the SLiCE method [73]. For the protein expression, *Escherichia coli* DH5α cells (New England Biolabs) were transformed with a prepared construct. Routinely, to ensure that the selected clones harbour the correct construct, the colony polymerase chain reaction was carried out using primers specific to the T7 promoter and terminator (T7-fwd and T7_rev) that flanking insertion site (Table S1). The reaction was performed using GoTaq^®^ Green Master Mix (Promega, Madison, WI, USA), and products were analysed by agarose gel electrophoresis. After confirmation, the construct was transformed into chemically competent *E. coli* BL-21 (DE3) (New England Biolabs) cells. *E. coli* BL-21 (DE3) cells harbouring the expression vector were grown in 1 L of LB medium containing kanamycin (50 μg/mL) at 37 °C. When OD_600_ reached 0.5–0.6, protein expression was induced by the addition of IPTG to a final concentration of 0.1 mM. Afterwards, the cells were incubated overnight at 16 °C with shaking at 200 rpm. After overnight incubation, cells were harvested by centrifugation at 4000× *g* for 25 min at 4 °C and pellets were washed twice with washing buffer (20 mM Tris−HCl pH 8.0, 300 mM NaCl, 5 mM imidazole) and resuspended in lysis buffer (50 mM Tris−HCl pH 8.0, 300 mM NaCl). The cells were disrupted with a sonicator (Bandelin, Sonoplus) and then centrifuged for 30 min at 4 °C and 9000× *g*. The recombinant protein was purified by nickel-based IMAC using cOmplete His-Tag Purification Resin (Roche) preequilibrated with washing buffer (20 mM Tris−HCl pH 8.0, 300 mM NaCl, 20 mM imidazole). A column with loaded protein was washed with a washing buffer, and expressed protein was eluted with elution buffer (20 mM Tris−HCl pH 8.0, 300 mM NaCl). The eluted fractions were concentrated to around 1.5 mL volume using an Amicon^®^ Ultra-15 Centrifugal Filter Unit (Merck KGaA, Darmstadt, Germany) with a 10 kDa cut-off. To check the purity of the obtained protein and molecular mass estimation, the SDS-PAGE analysis was performed. The protein concentration was measured by UV absorbance at 280 nm using a Take3 microvolume plate and a Biotek Synergy HT plate reader (BioTek, Santa Clara, CA, USA).

### 3.2. Operational Properties of Recombinant AlgL

#### 3.2.1. Determination of the Activity of Alginate Lyase

For the determination of the activity of AlgL, sodium alginate (Fluka, Buchs, Switzerland) at a concentration of 0.2% (*w*/*v*) in 50 mM phosphate buffer (pH 7.0) was used as a substrate. A total of 290 μL heated to 30 °C of substrate solution was transferred to 96-well UV transparent polystyrene microtiter plates (Greiner, Frickenhausen, Germany), and 10 μL of enzyme solution was added before starting the reaction. Next, the absorbance change at 235 nm for 5 min with 30 s intervals was measured using Tecan Infinite 200 Pro microplate reader at 30 °C. One unit of AlgL was an increase in 1.0-unit absorbance at 235 nm per minute per mL of 0.2% sodium alginate solution at assay condition. The enzyme activity was expressed as units per mg protein. The results were the average of three biological replicates and three technical replicates.

#### 3.2.2. Temperature Optimum and Thermal Stability Determination

The temperature optimum of AlgL was measured at 30, 40, 50, 60, 70, and 80 °C. Prior to adding, the substrate solution was incubated at given temperatures for 5 min to obtain suitable temperatures. The reaction of measurement of AlgL activity was carried out as mentioned above. The activity was expressed in relative terms, taking the highest activity at a given temperature of 100%. For determination of the thermal stability of the AlgL enzyme was stored at 4 °C and 37 °C for 30 days in 50 mM phosphate buffer (pH 7.0). The measurement of AlgL activity was carried out as mentioned above. The residual activity of AlgL was expressed in relative terms, taking initial activity as 100%.

### 3.3. Anti-Biofilm Activity Assays of AlgL

The biofilm inhibition and eradication were tested based on previously described methods with slight modification [9,74]. *P. aeruginosa* PAO-1 were obtained from the collection of the Department of Microbiology and Biotechnology of the West Pomeranian University of Technology in Szczecin. *P. aeruginosa* PAO-1 inoculum was prepared in TSB medium and incubated for 18 h at 37 °C on a shaker at 160 rpm. The inoculum was adjusted to 0.5 of the McFarland scale and then diluted 1:1000 in TSB medium supplemented with 1% glucose. For biofilm inhibition assay, 95 μL of diluted culture was transferred to sterile 96-well polystyrene microtiter plates, and 5 μL of alginate lyase at a final concentration of 0.8, 1.6, 3.2, 6.25, 12.5, and 25.0 μg/mL was added. The plates were incubated statically for 24 h at 37 °C. The nonadherent cells were removed by flushing three times in deionised water, and the biofilm was stained with 0.1% (*w*/*v*) crystal violet for 10 min. The excess of dye was removed by flushing with deionised water. Finally, the dye was solubilised in 96% (*v*/*v*) ethanol for 10 min., and the absorbance was measured by Tecan Infinite 200 Pro microplate reader at 595 nm. For biofilm eradication assay, 100 μL of *P. aeruginosa* PAO-1 culture was transferred to sterile 96-well polystyrene microtiter plates and incubated statically for 24 h at 37 °C. After incubation, the culture was gently removed, and the attached biofilm was rinsed three times in deionised water to remove nonadherent cells. Each well was filled with alginate lyase diluted in PBS buffer to a final concentration of 0.8, 1.6, 3.2, 6.25, 12.5, and 25.0 μg/mL and incubated at 37 °C for 1 h. Biofilm staining was prepared as described above. All experiments were carried out in triplicate.

### 3.4. AlgL Cytotoxicity Assay

To screen for any potential cytotoxicity of the recombinant AlgL enzyme towards mammalian subcutaneous cells, in vitro dose–response experiments were carried out using murine L929 fibroblasts, as described in our previous work [12]. Briefly, cells, passages 10–25, were maintained in Dulbecco’s Modified Eagle Medium (DMEM) containing 10% foetal bovine serum (FBS), 2 mM L- glutamine, 100 U/mL penicillin, and 100 μg/mL streptomycin. L929 cell line and all cell culture reagents were purchased from Merck (Poznań, Poland), while all plasticware was purchased from VWR (Gdańsk, Poland). For the dose–response assays, two 96-well plates were seeded in parallel: one with 10,000 cells per well for a 24 h experiment and one with 5000 cells per well for a 48 h experiment. After 24 h of culture, the media in both plates was aspirated and replaced with serial, threefold dilutions of recombinant AlgL or vehicle control (PBS) (4–6 technical replicates). The enzyme was prepared as a sterile, 2 mg/mL stock in PBS and was diluted 1:8 with media to yield the highest tested dose, 250 μg/mL. After incubation for 24 or 48 h, cell viability was assessed using an inverted light microscope (Delta Optical IB-100, Mińsk Mazowiecki, Poland) and resazurin cell viability assay [75]. 

### 3.5. Preparation of BC

The BC production was conducted as previously described [76]. Hestrin–Shramm (HS) (glucose 20 g/L, yeast extract 2.0 g/L, peptone 2 g/L, citric acid 1.15 g/L, Na_2_HPO_4_ 2.7 g/L, and MgSO_4_ × 7H_2_O 0.06 g/L supplemented with 1% ethanol and 0.01% of silicone polyether surfactant) medium was inoculated with 1-week-old starter culture of *K. xylinus* ATCC 53524. The cultivation was carried out in 24-well plates at 28 °C for 4 days. Following cultivation, the synthesised BC membranes were collected and rinsed in dH_2_O for 24 h. The next day, BC membranes were digested with 0.1 M NaOH at 80 °C (3×) to remove bacterial cells and residual medium components. Finally, BC membranes were rinsed again in dH_2_O until the pH was stabilised and sterilised by autoclaving at 121 °C for 15 min. The prepared BC membranes were stored at 4 °C before further analysis.

### 3.6. Zeta Potential of BC Membranes Determination

The isoelectric point (pH(I)) and pH-dependent zeta potential of the BC membranes was measured using SurPASS™ 3 analyser (Anton Paar GmbH, Graz, Austria). The 0.001 M KCl solution in ultrapure water was applied as an electrolyte, while the pH was adjusted using HCl and KOH standardised solutions.

### 3.7. Adsorption Experiment

The adsorption of AlgL on BC nanofibers was established according to [12] with slight modification. AlgL in concentrations from 0.2 mg/mL to 0.035 mg/mL were prepared in 50 mM phosphate buffer (pH 7.5). 5 mL of enzyme solution were incubated with ~0.035 g of wet BC membranes for 240 min at 25 °C with mixing. In the first hour, 100 μL samples were taken every 10 min. Then, the samples were collected every hour. Protein concentration was measured using a microplate reader with Greiner UV-star 96 well plates at 280 nm and calculated according to Lambert–Beer’s Law using molecular extinction coefficient (67,630 (M^−1^cm^−1^)):

(1) $$Q_{e}=\frac{\left(C_{0}-C_{e}\right)*V}{W}$$

where *C*_0_ is the initial and equilibrium concentration of AlgL (mg/mL), *C_e_* is the equilibrium concentration of AlgL (mg/mL), *V* is the volume of solution (mL), and *W* is the wet mass of BC membranes (g). 

### 3.8. Minimal Inhibitory Concentration (MIC) Determination

To determine MIC, doubling serial dilutions of gentamicin in MHB (Mueller Hinton Broth) were set up in 96-well microtiter plates, starting with 100 μL gentamicin and 100 μL *P. aeruginosa* PAO-1 inoculum adjusted to 0.5 of the McFarland scale and then diluted 1:100 in MHB. The positive control consisted of 100 μL inoculum and 100 μL MHB, while the negative controls consisted of 100 μL MHB only. Each set of tests and controls was set up with three replicates. The microtiter plate was incubated for 24 h at 37 °C. After incubation, the optical density of the microbial growth was determined at 600 nm using a microplate reader Tecan Infinite m200pro. The results are expressed as the average of three replicates. The MIC value was defined as the concentration of the ionic liquid that inhibited the growth of the microorganism by at least 90%.

### 3.9. Biofilm Development Analysis on BC Membranes with Immobilised Alginate Lyase

The ability of *P. aeruginosa* PAO-1 to grow and form biofilm on BC with immobilised AlgL was analysed according to [12,77]. To analyse the changes in the biofilm matrix on BC membranes, attenuated total reflectance Fourier transform infrared (ATR-FTIR) spectroscopy was used. BC samples with formed biofilm on their surface were dried at room temperature for 24 h before analysis. The analysis was conducted using an FTIR spectrophotometer ALPHA II (Bruker Co., Ettlingen, Germany) with a diamond ATR adapter. The spectra were collected in the range 4000–400 cm^−1^ with a resolution of 2 cm^−1^ (32 scans). The obtained ATR-FTIR spectra were analysed using SpectraGryph 1.2 software. From each spectrum, the baseline was removed, and then spectra were normalised to the area of the band at the amide II region (≈1530 cm^−1^). The preprocessed spectra were used for 2D correlation spectroscopy analysis using OriginPro2021. Next, the significant cross bands were selected and evaluated according to the ratio between the area of bands depending on the amount of immobilised AlgL.

### 3.10. Assessment of Antibiotic Susceptibility by Flow Cytometry Method

AlgL in a concentration of 0.2 mg/mL and gentamicin sulphate salt (Merck KGaA, Darmstadt, Germany) in a concentration in the range of 0.25–1.0 μg/mL were immobilised on BC membranes, as mentioned above. Next, the overnight culture of *P. aeruginosa* PAO-1 was normalised to 0.5 MacFarland scale and diluted 1:100 in TSB medium supplemented with 1% of glucose. Following the 50 μL of prepared bacterial suspension was spread on the surface of BC membranes with immobilised AlgL and gentamicin and incubated at 37 °C for 24 h. After incubation, BC membranes were rinsed in 50 mM phosphate buffer (pH 7.4) to remove the nonadherent cell, rinsed again in 50 mM phosphate buffer (pH 7.4) and vortexed three times for 1 min. Next, the solution with released bacterial cells was centrifuged for 10 min at 6000 rpm and rinsed in 1 mL of 50 mM phosphate buffer (pH 7.4). The live/dead staining was done using SYTO 9 (Thermo Fisher Scientific, Waltham, MA, USA) and propidium iodide (Thermo Fisher Scientific) according to the manufacturer’s instructions. The number of live and dead bacterial cells released from one BC membrane was assessed using a BD Accuri C6 Plus flow cytometer.

### 3.11. Statistical Analysis

Statistical analyses were conducted using OriginPro2021. The statistical significance differences of means were tested by Tukey’s multiple comparison test, and statistical significance was considered with *p* < 0.05.

## 4. Conclusions

Bacterial cellulose has been found to be an ideal material for wound dressing applications. In its wet state, it meets all the conditions that an ideal dressing should have, such as high tensile strength, flexibility, good water-holding capacity, biodegradability, and biocompatibility. Moreover, the highly porous structure of BC, due to the presence of numerous hydroxyl groups, allows for the immobilisation of various compounds on its surface, giving BC new unique properties [41,78]. In this work, the potential of BC membranes as a carrier for simultaneous immobilisation of AlgL purified from *P. aeruginosa* PAO-1 and gentamicin for wound dressing applications was investigated. The use of alginate, an enzyme that catalysed degradation of alginate, an essential component of the biofilm matrix of mucoid *P. aeruginosa* strains, resulted in the inhibition of exopolysaccharides formation by a tested strain of *P. aeruginosa* PAO-1 on BC membranes. This allowed the gentamicin present in the dressing to be more effective against *P. aeruginosa* cells, even at a very low dose. To sum up, this innovative dressing material may be a solution to the widespread overuse of antibiotics and, consequently, the problem of antibiotic resistance that has been growing for years. 

## Figures and Tables

**Table 1:** Langmuir and Freundlich isotherms constants and values of R^2^ for the adsorption of AlgL on BC membranes.

Langmuir Isotherm			Freundlich Isotherm		
b (L/mg)	q_m_ (mg/g)	R^2^	K_f_ (L/g)	1/n	R^2^
0.009	45.79	0.9984	1.28	0.62	0.9596

b—Langmuir constant, q_m_—maximum adsorption capacity, K_f_—Freundlich isotherm constant, 1/n—adsorption intensity.

## Data Availability

The data presented in this study are available on request from the corresponding author.

## Supplementary Materials

The following supporting information can be downloaded at https://www.mdpi.com/article/10.3390/ijms24054740/s1.
