# Supplementary material for: Enhancement of Inhibition of the Pseudomonas sp. Biofilm Formation on Bacterial Cellulose-Based Wound Dressing by the Combined Action of Alginate Lyase and Gentamicin
Source: Int J Mol Sci. 2023 Mar 1;24(5):4740. doi: 10.3390/ijms24054740 (PMC10002595; doi:10.3390/ijms24054740)
Supplement: Supplementary file 1 [file ijms-24-04740-s001.zip › ijms-2233018-supplementary.pdf]

Enhancement of inhibition the Pseudomonas sp., biofilm formation on bacterial cellulose based wound dressing by combined action of alginate lyase and gentamicin

Magdalena Szymańska[1], Katarzyna Przygrodzka[1], Anna Żywicka[1], Bartłomiej Grygorcewicz[2], Piotr Sobolewski[3], Sylwia Mozia[4], Marcin Śmiglak, Radosław Drozd[1]

[1]Department of Microbiology and Biotechnology, Faculty of Biotechnology and Animal Husbandry, West Pomeranian University of Technology in Szczecin, 45 Piastow Avenue, 71-311, Szczecin, Poland

[2]Department of Laboratory Mediine, Chair of Microbiology, Immunology and Laboratory Medicine, Pomeranian Medical University in Szczecin, 72 Powstańców Wielkopolskich Str., 70-111, Szczecin, Poland

[3]Department of Polymer and Biomaterials Science, Faculty of Chemical Technology and Engineering, West Pomeranian University of Technology in Szczecin, 45 Piastow Avenue, 71-311, Szczecin, Poland

[4]Department of Inorganic Chemical Technology and Environment Engineering, Faculty of Chemical Technology and Engineering, West Pomeranian University of Technology in Szczecin, ul. Pułaskiego 10, 70-322 Szczecin, Poland

[5]Poznan Science and Technology Park (PPNT), Rubiez 5, 61-612 Poznan, Poland

Table S1. Oligonucleotides for PCR amplification and colony-PCR.

Primer	Sequence (5'to 3')
AlgL_fwd	cctggtgccgcgcggcagccatATGGCCGACCTGGTACCCCCGCC
AlgL_rvs	aagcttgtcgacggagctcgaattcTCAACTTCCCCCTTCGCGGC
pET28a_fwd	tgagatccggctgctaacaaagc
pET28a_rvs	catatggctgccgcgcgg
T7_fwd	TAATACGACTCACTATAGGG
T7_rvs	GCTAGTTATTGCTCAGCGG

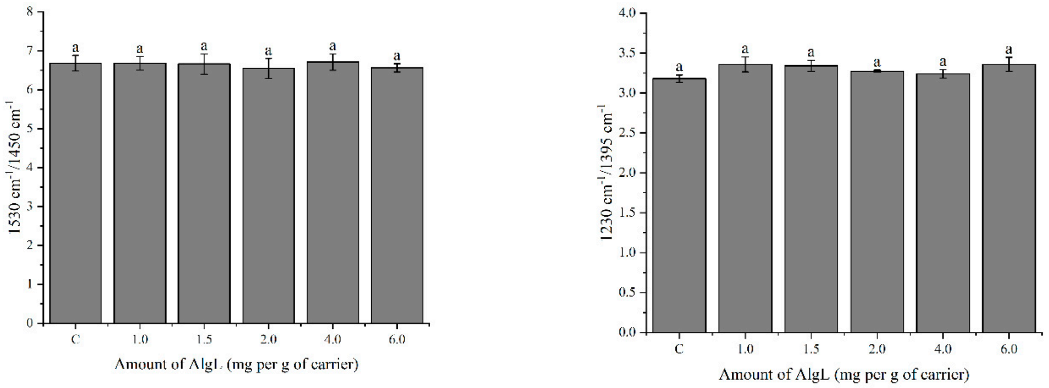

(a) (b)

Figure S1. The relative changes in the band ratio 1530 cm^{-1}/1450 cm^{-1} (a) and 1230 cm^{-1}/1395 cm^{-1} (b). The means with the same superscript are not significant different with p *>0.05*. Error bars represent standard deviation.

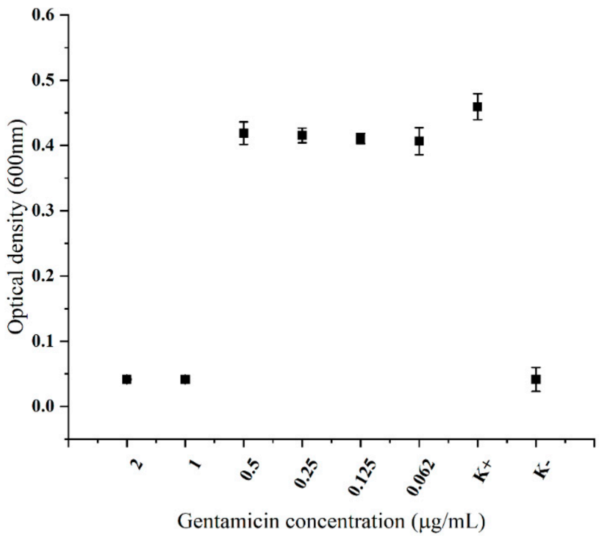

Figure S2. Minimal inhibitory concentration (MIC) of gentamicin for *P. aeruginosa* PAO-1. K- is a negative control, and K+ is a positive control.
